# Operative vs conservative treatment in distal radius fractures: A protocol

**DOI:** 10.1097/MD.0000000000021250

**Published:** 2020-07-17

**Authors:** Meng Wu, Xiongfeng Li, Jianyou Li, Yonghua Chen

**Affiliations:** a Department of Orthopedics and Trauma, Huzhou Central Hospital and Affiliated Central Hospital of Huzhou University; b Department of Orthopedics and Trauma, Huzhou Traditional Chinese Medicine Hospital Affiliated to Zhejiang Chinese Medical University, Zhejiang Province, China.

**Keywords:** casting, distal radius fractures, open reduction internal fixation, procotol

## Abstract

**Background:**

Given this lack of conclusive outcome data, there are currently no clear guidelines to direct the treatment of displaced distal radius fractures in the elderly. This retrospective clinical trial was performed to compare the outcomes of two methods that were used for the treatment of displaced and unstable distal radial fractures in patients 65 years of age or older:

(1) open reduction internal fixation with use of a volar locking plate and
(2) closed reduction and plaster immobilization (casting).

**Methods:**

This study was performed and reported in accordance with the Strengthening the Reporting of Observational studies in Epidemiology checklist. Between January 2017 and May 2018, a total of 184 patients who presented to Huzhou Traditional Chinese Medicine Hospital with distal radius fractures were extracted from the hospital database and evaluated for eligibility. This retrospective cohort study was approved by the institutional review board in our hospital. Outcome measures included Patient-Related Wrist Evaluation score, patient satisfaction, complications, and radiographic outcomes. SPSS software package (version 21.0; SPSS Inc, Chicago, IL) was used for all statistical analyses.

**Results:**

The hypothesis was that the two groups would achieve similar functional scores and complications in distal radial fractures.

**Trial registration:**

This study protocol was registered in Research Registry (researchregistry5689).

## 1 Introduction

Distal radius fractures (DRFs) are the second most prevalent fracture in elderly individuals and affect more than 85,000 older Americans each year.^[1,2]^ DRFs are associated with substantial increases in healthcare consumption. In the 6-month period following a DRF, the average Medicare beneficiary incurs $7700 more in charges relative to prefracture levels.^[3,4]^ Annually, DRFs cost $535 million in direct medical expenses alone.^[5,6]^ The fractures in different age groups need to be treated differently and properly. When displaced DRFs occur in young active patients, open reduction and internal fixation is commonly selected as the definitive treatment.^[7,8]^ However, when similar fractures occur among the elderly, the most appropriate form of management is less clear due to a number of factors, such as decreased functional demands, higher perioperative risks, poorer bone quality, etc.^[9,10]^

Both conservative (such as casting) and operative treatments (such as open reduction internal fixation, external fixation, intramedullary fixation, or percutaneous pinning) had been advocated as successful treatments for distal radius fractures. Casting is noninvasive but malunion or fracture collapse can ensue. Although several surgical options are available, the 2009 American Academy of Orthopedic Surgeons clinical practice guideline was unable to recommend for or against any one specific surgical method.^[11]^ Despite this lack of consensus, open reduction internal fixation of distal radius fractures has become increasingly popular in recent years, particularly in relation to the use of volar locking plates.^[12–15]^

There had been a few studies of operative versus conservative treatment of distal radius fractures in the elderly published recently, but the results were not conclusive.^[5,16–18]^ A previous meta-analysis showed that surgery and non-surgical treatment both had their own advantages and disadvantages.^[19]^ A recently published meta-analysis revealed that no significant differences in most functional assessments were found when comparing surgical and nonsurgical management of distal radius fractures. Thus the authors concluded that nonsurgical treatment for the distal radius fractures should be considered firstly and indications for operative fixation should be considered carefully in the treatment of distal radius fractures.^[20]^

Given this lack of conclusive outcome data, there are currently no clear guidelines to direct the treatment of displaced distal radius fractures in the elderly. This retrospective clinical trial was performed to compare the outcomes of two methods that were used for the treatment of displaced and unstable distal radial fractures in patients 65 years of age or older:

(1) open reduction internal fixation with use of a volar locking plate and
(2) closed reduction and plaster immobilization (casting). The hypothesis was that the two groups would achieve similar functional scores and complications in distal radial fractures.

## 2 Materials and methods

### 2.1 Patients

This study was performed and reported in accordance with the Strengthening the Reporting of Observational studies in Epidemiology checklist. Between January 2017 and May 2018, a total of 184 patients who presented to Huzhou Traditional Chinese Medicine Hospital with distal radius fractures were extracted from the hospital database and evaluated for eligibility. This retrospective cohort study was approved by the institutional review board in Huzhou Traditional Chinese Medicine Hospital (ZDHTCM001484) and was registered in the Research Registry (researchregistry5689). Patients included in this retrospective study were age ≥65 years and had a distal fracture of the radius, AO classification type A or C. Type C fractures had no stepoff or gap deformity of the articular surface. Exclusion criteria were age < 65 years, oblique fractures (AO classification type B), pathological fractures, open fractures, volar angulated fractures (Smith fracture), patients presenting more than a week after injury, patients with dementia or psychiatric illness, bone and joint diseases that could interfere with rehabilitation, past ipsilateral upper limb surgery or trauma, accompanying other bone and/or soft tissue injuries (including carpal bones), bilateral fractures, and fractures treated with open reduction and plate or pin fixation.

### 2.2 Surgical techniques

In the casting group, all fractures were initially treated with closed reduction and immobilization in a dorsoradial plaster cast. Patients were treated with a closed forearm cast in a neutral position for 6 weeks. The active digital range of motion was started immediately. After cast removal, physiotherapy was started. The protocol permitted conversion to secondary surgical treatment in the case of significant loss of reduction or pronounced joint incongruence.

In the internal fixation group, all operations were performed with the patient in the supine position under general anesthesia. No nerve blocks were performed on all patients to help control postoperative pain. Patients were treated primarily or after soft-tissue conditioning by open reduction with volar locking plate fixation via the volar Henry approach. The protocol permitted the use of implants from any manufacturer according to local standards and depending on availability. All surgical procedures were performed by experienced hand surgeons. No additional bone grafting was used. After surgery, the wrist was immobilized in a below-the-elbow splint for pain reduction. The active digital range of motion was started immediately. Ten to twelve days after surgery, the sutures were removed and the wrist was placed in a removable splint for another week. At that time, physiotherapy with active and passive wrist mobilization out of the splint was started.

### 2.3 Outcome evaluation

Outcome measures included Patient-Related Wrist Evaluation (PRWE) score, patient satisfaction, complications, and radiographic outcomes (Table 1). The PRWE score is a 15-item questionnaire composed of 3 subscales: pain, specific activities, and usual activities. The total score of the PRWE, including all 3 subscales, can range from 0 (no pain or disability) to 100 (maximal pain or disability). Patient satisfaction was assessed by asking the question “How satisfied are you with your wrist?” one year after surgery. The response was recorded using a 5 point Likert scale: very satisfied, satisfied, neutral, unsatisfied and very unsatisfied. Patients who recorded very satisfied or satisfied were classified as satisfied. For patients who were not seen recently, the scores were obtained via telephone. An adverse event was defined as any event that necessitated another surgical intervention or additional medical treatment.

**Table 1:**
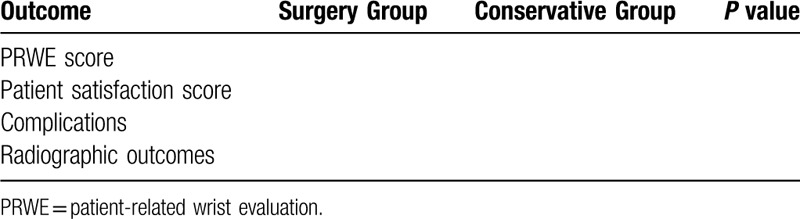
Postoperative outcomes.

Complications included loss of reduction, fracture malunion, and fracture nonunion as well as deep infection, neuropathy, tendon irritation, and tendon rupture. Complex regional pain syndrome was diagnosed on the basis of the presence of dysesthetic pain and hyperesthesia extending into the hand of the injured limb, vasomotor changes, skin atrophy, and diffuse osteopenia. Post-operative complications and revision procedures were documented during routine collection of follow-up data. All data were independently verified by a detailed review of hospital operative reports, anesthesia records, and clinical records.

Radiographic outcomes with regard to fracture union, loss of reduction, and development of arthritis was assessed at each visit. Measurements of radial inclination, radial height, tilt, ulnar variance, and articular step-off were made on each radiograph by a trained research associate under the direction of the treating surgeon. Arthritic change or its advancement was noted if present, with use of the system described by Knirk and Jupiter, at the 3, 6, and 12-month examinations.

### 2.4 Statistical analysis

The Student *t* test and the Mann-Whitney *U* test were used to compare age, duration of symptoms, follow-up period, PRWE score, patient satisfaction, and radiographic outcomes between the 2 groups. Fisher exact test and the Chi-square test were used to compare gender, affected wrist, smoker, and complications between the 2 groups. Significance was set at a level of 0.05 with 95% confidence intervals. SPSS software package (version 21.0; SPSS Inc, Chicago, IL) was used for all statistical analyses.

## 3 Discussion

The management strategies for DRFs are controversial. Many studies and systematic reviews performed in the past to determine the best management of DRFs in the elderly failed to reach a consensus partly due to variety of treatment options. One Cochrane review comparing external fixation and conservative treatment of DRFs in adults concluded that there was insufficient evidence to confirm a superior functional outcome, but external fixation did reduce displacement and provided improved anatomical results with only minor complications.^[21]^ Other Cochrane reviews examining surgical interventions and conservative interventions for DRFs concluded that there was insufficient evidence to determine when to perform surgery, or what type of surgical or nonsurgical management is best.^[22]^ A review specifically examining percutaneous pinning for DRFs found that the precise role and methods of percutaneous pinning have not been established, and that Kapandji pinning and biodegradable materials are often associated with a higher rate of complications.^[23]^

The limitations of our study included those inherent in any retrospective cohort study, including the possibility of selection or observational bias. This study also did not address long-term follow-up (5–10 years) as our study relied on electronic medical records kept since 2017. The authors recognize that longer term follow-up is critical in determining the influence of therapy on function outcomes.

## Author contributions

Meng Wu and Xiongfeng Li, planned the study design and wrote the study protocol. Jianyou Li reviewed the study protocol. Meng Wu, Xiongfeng Li, and Jianyou Li will recruit participants and collect data. Meng Wu and Yonghua Chen wrote the manuscript. Jianyou Li funded and supported this study. All of the authors have read, commented on, and contributed to the submitted manuscript.
